# The* In Vitro* and* In Vivo* Anticancer Properties of* Moringa oleifera*

**DOI:** 10.1155/2018/1071243

**Published:** 2018-11-14

**Authors:** Kang Zi Khor, Vuanghao Lim, Emmanuel J. Moses, Nozlena Abdul Samad

**Affiliations:** ^1^Integrative Medicine Cluster, Institut Perubatan dan Pergigian Termaju (IPPT), Sains@BERTAM, Universiti Sains Malaysia, 13200 Kepala Batas, Pulau Pinang, Malaysia; ^2^Regenerative Medicine Cluster, Institut Perubatan dan Pergigian Termaju (IPPT), Sains@BERTAM, Universiti Sains Malaysia, 13200 Kepala Batas, Pulau Pinang, Malaysia

## Abstract

*Moringa oleifera*, a fast-growing deciduous tree that is widely cultivated in tropical and subtropical regions of the world, is well known for its abundant uses. The tree is a source of food, shelter, and traditional medicine for many people, especially in developing countries. Many studies have been conducted to evaluate the various claims of traditional medicine practitioners that the moringa tree can improve health and treat various diseases. The tree has a high nutritional profile, especially the nutrient rich leaves. Some reports also support the use of parts of the tree to reduce blood sugar and cholesterol levels. These attractive properties have led researchers to look for other novel uses for the moringa tree, especially as a source of anticancer drugs. Researchers have tested extracts from various parts of the moringa tree both* in vitro* and* in vivo *on several types of cancers with varying success. This review explores the state of current research on the anticancer properties of* M. oleifera.*

## 1. Introduction

*Moringa oleifera*, better known as the drumstick tree or simply known as moringa, is native to the foothills of the Himalayan ranges in the Indian subcontinent [1]. The tree, called the ‘miracle tree' by some, is renowned for its abundant uses. It was first brought to the Mediterranean during the Graeco-Roman period and to Southeast Asia by Indian traders. Later the tree spread around the world with Indian migration, eventually reaching the African continent, the Caribbean islands, South America, and the Southern Pacific islands [2]. Indian migrants tried to grow the tree wherever they settled, and it provided them with food, shelter, and medicine. The moringa tree is very hardy and can grow in most tropical and subtropical climates. It requires minimal care, can withstand droughts, and is able to grow in poor soil. In the 21^st^ century, people are still promoting the growth of* M. oleifera* in regions with suitable climate. For example, the late revolutionary leader of Cuba, Fidel Castro, actively promoted the cultivation of the moringa tree in Cuba after his retirement from politics [3]. He even sent dignitaries to India to obtain seeds for different varieties to be brought back to Cuba for cultivation. Castro wrote that the tree ‘is the only plant that has every kind of amino acid. With proper planting and management, its green-leaf production can exceed 300 tonnes per hectare in a year. It has dozens of medicinal properties.' He personally grew the trees in his home garden to provide leaves and pods for his own consumption.

The main use of* M. oleifera* is as a food source. Its pods are favoured by Indians for use in curries. Special breeds known as ‘PKM-1' and ‘PKM-2' were developed by Tamil Nadu University in India specifically to produce pods [4]. These specialty breeds can produce pods six months after planting as compared to regular varieties, which take a year to produce pods. The leaves of the moringa tree are commonly eaten as a vegetable in India, Southeast Asia, and Africa. Mature leaves are not fibrous and are suitable for stir frying. The leaves can also be dried, ground into a fine powder, and used as a supplement by adding small amounts to soup, bread dough, and stews. Moringa leaf powder has been promoted as a health food in many countries due to its high nutritional profile. It has been suggested that leaf powder could be added to food aid provided by the UN to enrich food with vital nutrients and that moringa trees should be planted in areas with low food security as a buffer against malnutrition [5].

The seeds of* M. oleifera* are also edible. Moringa seeds are high in oil content and can be pressed to extract the oil. Moringa seed oil, which is known commercially as ben oil, has the nutritional profile and characteristics of olive oil [6] and can be used in lieu of olive oil. Ben oil also is used commercially in the production of cosmetics and perfumes due to its stability [7]. Ben oil is very rich in antioxidants and is therefore very stable and does not turn rancid for years. Pressed seed cakes from which the oil has been extracted are still rich in nutrients and can be used as animal fodder or as plant fertilizer. Seed cakes can also be used as a flocculating agent for water treatment. They can be added to turbid water containing suspended solids, and they will clump the solids together and clarify the water. Moringa seed cakes are a viable alternative to conventional flocculating agents such as aluminum sulfate [8]. They have a marked advantage over chemical agents because they are natural, biodegradable products with antimicrobial properties [9]. As seed cakes are a by-product of oil extraction, they have minimal production costs and offer added value to producers who can turn a waste product into a usable product.

Moringa trees have big and deep tap roots that enable them to grow in areas with low rainfall. It is very hardy and adaptable to tropical and subtropical climates. It can tolerate low water environments and requires minimal care to grow well, and it is common to see moringa trees planted as shade trees beside municipal roads in India and Southeast Asia. It is found in gardens in Middle Eastern countries such as Saudi Arabia and the United Arab Emirates, as it can grow in arid regions with minimal water [10]. The trees can also be grown as living fences in gardens or farms.* M. oleifera* is a good candidate for agroforestry projects in tropical and subtropical climates due to its ease of cultivation and multiple uses [11]. They can be used as a wind break and to prevent soil erosion. They are especially suitable for planting as a wind break at desert edges, to prevent desertification, and in attempts to reclaim areas that have already been turned to desert. Thus, the moringa tree provides ecological services.

**Figure 1:**
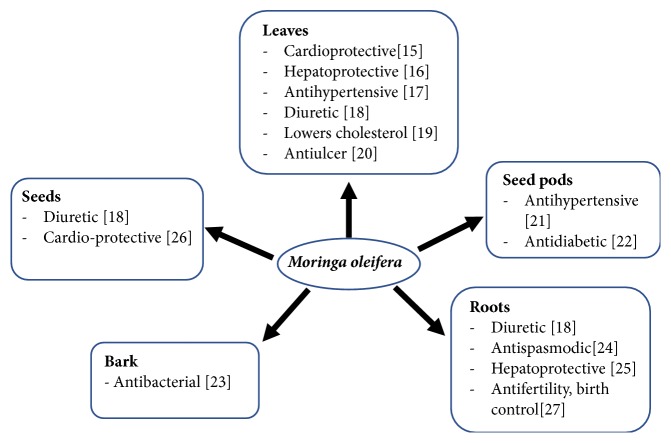
Medicinal uses of* Moringa oleifera* (see [15–27]).

The various parts of* M. oleifera* are used extensively in traditional medicine in many regions of the world. In its native land of India, it is used in ayurvedic medicine and is believed to be able to prevent 300 diseases [12]. It is used for varied functions such as cleansing the blood and liver, strengthening the heart, increasing fat metabolism to promote weight loss, and even removing worms [13]. In other regions of prevalent moringa cultivation, such as Southeast Asia and South Asia, different parts of the moringa plant are thought to have antidiabetic, antibacterial, antitumor, antipyretic, antiepileptic, anti-inflammatory, antiulcer, antispasmodic, diuretic, antihypertensive, cholesterol lowering, antioxidant, hepatoprotective, antibacterial, and antifungal activities [14].  Figure 1 shows some of the medicinal uses of the different parts of the moringa plant. Some of these medicinal uses have been verified by researchers. For example, in a study on the ability of the leaf extract to prevent isoproterenol-induced myocardial damage in Wistar albino rats, Nandave et al. (2009) [15] found that the extract significantly reduced lipid peroxidation in myocardial tissue, thus confirming the cardioprotective effect and antioxidant activity of* M. oleifera. *Pari and Kumar (2002) [16] described the hepatoprotective effects of moringa leaf extract against liver damage in rats induced by the antitubercular drugs isoniazid, rifampicin, and pyrazinamide. Blood test results and histopathological examination of liver sections showed that rats treated with moringa leaf extract recovered better from hepatic damage induced by antitubercular drugs than did untreated rats. These findings support the use of moringa for some treatments used in traditional medicine. However, scientific interest in the antitumor/anticancer activities of* M. oleifera* has been growing due to the increasing incidence of cancer, and this will be the main focus of our review.

## 2. Natural Products as Anticancer Agents

Based on statistics published by the World Health Organization, cancer is the second leading cause of death worldwide, causing an estimated 8.8 million deaths [28]. Cancer incidences are expected to rise by approximately 70% in the next 20 years. About 70% of cancer deaths occur in low- and middle-income countries, likely because of factors such as increasing pollution levels, increased life expectancy, insufficient healthcare facilities, and expensive anticancer drugs. One way to overcome these challenges is to develop anticancer drugs from natural sources such as plants, which might lead to more affordable drugs for low- and middle-income countries. Plants and natural resources such as marine organisms and microorganisms are among the major sources of anticancer drugs. More than 60% of all current anticancer drugs are derived from natural sources [29]. As an example, the anticancer drug camptothecin is derived from the extract of the plant* Camptotheca acuminata *and its chemical derivatives topotecan and irinotecan [30]. Compared to conventional drugs, plant-derived phytochemicals have fewer adverse effects and lower toxicity, which has led to intensified research on medicinal plants [31].* M. oleifera* holds great potential as a source of anticancer drugs due to its low toxicity [32] and the presence of a variety of phytochemicals [33]. There has been extensive interest in exploring the anticancer activities of various parts of the* M. oleifera* tree, and many published research articles describe promising results of* in vitro* and* in vivo* testing of various extracts from the moringa plant.

## 3. Moringa Leaf Extract: A Potential Anticancer Agent

All parts of the* M. oleifera* tree have been tested for anticancer activity, including the leaves, seeds, bark, and roots. However, the most extensive research on the anticancer activities of* M. oleifera* has focused on the leaf extracts. The moringa tree is an evergreen that grows new leaves year-round, with a projected production of six tons per hectare per year [34]. The leaves are rich in polyphenols and polyflavonoids, which are antioxidants and potential anticancer compounds [35].

Many researchers start by exploring the antioxidant activity and anti-inflammatory activity of the leaf extracts as a preliminary screening for anticancer activity. One of the factors that causes cancer is oxidative stress which is an imbalance in production of free radicals and oxidants and their elimination by antioxidants [36]. Antioxidants can disrupt the formation of free radicals and reduce oxidative stress, which ultimately prevents cancer. The next step often involves testing the effects of the leaf extracts* in vitro* on cancer cell lines by examining the extract's impact on growth and proliferation of cancerous cells and on cell morphology. If the leaf extract shows promising anticancer activity for a specific cancer cell line, the researchers usually proceed by identifying the specific pathways disrupted by the extract through molecular analysis. With sufficient evidence, some researchers will continue exploring the anticancer activity of the extract* in vivo*, usually in mouse or rat models, to observe the actions of the leaf extract in a living mammal, which is a more accurate representation of the human body. The articles reviewed herein will focus on research showing the antioxidant and anticancer activities of moringa leaf extracts* in vivo* and* in vitro* and the pathways/mechanisms of action through which these effects may occur.

### 3.1. Antioxidant Activities

Verma et al. (2009) [37] explored the antioxidant activity of various fractions of moringa leaf extracts. They started by exhaustively extracting moringa leaf powder over three days with 50% methanol. The crude extract was partitioned to obtain four additional fractions. The five fractions produced were the crude extract, diethyl ether extract, phenolic fraction, polyphenolic fraction, and aqueous fraction. The five fractions were tested for their antioxidant activities using several different antioxidant test models because not all compounds will react in the same way in different antioxidant test models [38]. The fractions were tested with eight different antioxidant tests: *β*-carotene bleaching and linoleic acid assay, ferric-reducing antioxidant power assay, free radical scavenging activity measured with 2,2-diphenyl-1-picryl-hydrazyl-hydrate (DPPH), superoxide anion radical scavenging activity, non-site-specific hydroxyl radical scavenging assay, site-specific hydroxyl radical scavenging assay, ferrous ion chelating capacity, and lipid peroxidation. All eight assays gave consistent results and showed that the polyphenolic fraction had the highest antioxidant activity, followed by the crude extract, diethyl ether fraction, nonphenolic fraction, and finally the aqueous fraction with the weakest antioxidant activity.

Once the polyphenolic fraction was determined to have the highest antioxidant activity, another test to determine the polyphenolic fraction's ability to prevent DNA nicking was conducted. The supercoiled plasmid DNA was incubated with H_2_O_2_ and different concentrations of the polyphenolic fraction and then analyzed with agarose gel electrophoresis. The results showed that 10 *µ*g/ml of the polyphenolic fraction provided comparable protection to DNA as 5U of catalase (an enzyme that breaks down H_2_O_2_) and 50 *µ*M of quercetin (a strong antioxidant). The results from the antioxidant test and DNA nicking prevention test suggest that the polyphenolic fraction is a strong antioxidant. Based on these results, Verma et al. (2009) [27] proceeded to conduct* in vivo* testing of antioxidant activity using Sprague-Dawley rats treated with carbon tetrachloride (CCl_4_), resulting in increased oxidative stress [39]. They found that the polyphenolic fraction could reverse many of the ill-effects of CCl_4_, including elevated levels of lipid peroxides (markers for oxidative stress) and low levels of glutathione (GSH), catalase (CAT), and superoxide dismutase (SOD), which are strong antioxidants. The antioxidant activity of 100 mg/kg of the polyphenolic fraction was comparable to the antioxidant activity of 50 mg/kg of vitamin E. The researchers also identified the active compounds which contribute to the antioxidant activity which are mainly phenolic acids and flavonoids. In summary, this study showed that moringa leaf extract is a source of strong antioxidants with antioxidant activity both* in vitro* and* in vivo*, which indicates that it has potential anticancer activity.

In another study, Sreelatha and Padma (2011) [40] explored the modulatory effect of moringa leaf extracts against H_2_O_2_-induced cytotoxicity and oxidative damage in the HeLa-derived KB cell line. They also compared activity between extracts from tender and mature leaves. Tender and mature leaves were extracted with hot water, and the phenolic composition was measured using high performance thin layer chromatography (HPTLC). Quercetin and kaempferol were the main phenolic compounds present, with levels of quercetin at 795 and 436.2 *µ*g/ml in mature and tender leaves, respectively, and levels of kaempferol at 216 and 115 *µ*g/ml in mature and tender leaves, respectively. Mature leaves contained almost twice the amount of phenolic compounds as tender leaves, suggesting that mature leaves might have higher antioxidant activity than tender leaves. The antioxidant activity of the two leaf extracts was measured using the *β*-carotene bleaching and linoleic acid assay. As expected, the mature leaf extract had higher antioxidant activity (84.6% of the activity of *β* -carotene) compared to that of the tender leaf extract (63.6%). These extracts were then tested for their effects against H_2_O_2_-induced cytotoxicity and oxidative damage in the KB cell line. Single cell electrophoresis to detect DNA damage in KB cells showed significant reduction in DNA damage for cells treated with both mature and tender leaf extracts when compared to untreated cells. These results indicate that moringa leaf extract can prevent H_2_O_2_-induced DNA damage in KB cells.

Sreelatha and Padma (2011) [40] then tested whether the leaf extracts could prevent cytotoxic effects on H_2_O_2_ treated cells using the 3-(4,5-dimethylthiazol-2-yl)-2,5-diphenyltetrazolium bromide (MTT) assay to determine cell viability. Mature leaf extracts were slightly better at modulating the effects of H_2_O_2_ (i.e., higher cell viability) compared to tender leaf extracts. However, treatment of KB cells with the leaf extracts alone also reduced cell viability of KB cells by 20–40%. This suggests that the leaf extracts have cytotoxic effects on KB cells in the absence of H_2_O_2_ but when used in conjunction with H_2_O_2_ they reduce H_2_O_2_-induced cytotoxicity. The researchers next tested the lipid peroxide levels in H_2_O_2_-exposed KB cells and cells treated with leaf extracts. The KB cells were treated and the levels of malondialdehyde (MDA), which is the product of lipid peroxidation and a good indicator of oxidative stress within cells, were measured. Cells treated with H_2_O_2_ had increased levels of MDA compared to untreated cells. Cells treated with H_2_O_2_ and leaf extract had lower levels of MDA compared to cells treated with H_2_O_2_ alone, indicating that the leaf extract reduced lipid peroxidation in H_2_O_2_-treated cells. There were no significant differences between the results for mature and tender leaf extracts. However, treatment with leaf extracts alone increased the MDA levels compared to the untreated control as well. In the next experiment, the levels of the antioxidant enzymes SOD and CAT were measured. KB cells treated with H_2_O_2_ showed a significant decrease in SOD and CAT levels, as the enzymes were used to remove the H_2_O_2_. Cells treated with H_2_O_2_ and then leaf extract showed less significant decreases in SOD and CAT levels. Cells treated with leaf extract alone did not show any significant decrease in SOD and CAT levels compared to untreated cells. The active compounds that provide these activities are quercetin and kaempferol. The results of the whole study indicate that moringa leaf extract is a good antioxidant scavenger, as it removes H_2_O_2_ and increases viability of KB cells exposed to H_2_O_2_. However, moringa leaf extract alone is cytotoxic to KB cells and reduces KB cell viability, but this effect is not caused by compromising SOD or CAT levels.

### 3.2. In Vitro Anticancer Activities

A number of studies have focused on the anticancer activity of the moringa leaf extract using* in vitro* screening of cancer cell lines. Parvathy and Umamaheshwari (2007) [41] studied the effects of moringa leaf extracts on the human B-lymphocyte plasmacytoma—U266B1 cell line. U266B1 cells were treated with serial dilutions of the methanol, ethanol, ethyl acetate, and chloroform extracts of the moringa leaf, and cytotoxicity was measured using the neutral red dye uptake assay. The methanol extract had the highest cytotoxic activity against U266B1 cells (IC_50_: 0.32 *µ*g/ml); this suggests that this extract has high anticancer activity, as a small amount can significantly inhibit the proliferation of U266B1 cells. In another study, Nair and Varalakshmi (2011) [42] tested the anticancer and cytotoxic potential of hot water, methanol, and hexane extracts of the moringa leaf on cervical cancer cells (HeLa cell line) and normal lymphocytes. Results of the MTT assay showed that the aqueous leaf extract caused a dose-dependent decrease in HeLa cell viability (IC_50_: 70 *µ*g/ml). In contrast, the methanol and hexane leaf extracts caused an increase in HeLa cell viability at higher concentrations. Lymphocytes treated with the different leaf extracts did not exhibit any significant decrease in cell viability. The trypan blue dye exclusion assay was performed for the aqueous leaf extract to verify the results from the MTT assay. The results of this experiment also showed a dose-dependent decrease in cell viability for the aqueous leaf extract. The HeLa cells exhibited increased numbers of detached and dead cells with increasing concentration of the aqueous leaf extract. DNA fragmentation analysis of cells treated with the aqueous leaf extract showed a DNA smear on the agarose gel compared to distinct bands for untreated cells. The DNA smear is indicative of DNA breakage and damage. The last test performed was acridine orange-ethidium bromide staining, which can distinguish viable cells (green fluorescence glow) from nonviable cells (bright orange chromatin). These results showed that the aqueous leaf extract of moringa leaves has significant cytotoxic activity and is capable of fragmenting DNA and killing the HeLa cells.

Charoensin (2014) [43] compared the anticancer activity of the leaf extract among different cancer cell lines (hepatocarcinoma (HepG2), colorectal adenocarcinoma (Caco-2), and breast adenocarcinoma (MCF-7)). First, the methanol and dichloromethane leaf extracts of* M. oleifera* were tested for their antioxidant activity using the DPPH and 2,2'-azino-bis (3-ethylbenzothiazoline-6-sulphonic acid) assays. The results of both assays indicated that the methanol extract had better antioxidant activity. The anticancer activity of the leaf extracts was then tested on the three cell lines. The MTT assay, conducted to assess the effects on cell proliferation, showed that the dichloromethane leaf extract (IC_50_: 112–133 *µ*g/ml) was more effective than the methanol leaf extract (250 *µ*g/ml) at killing cancer cells. This means that a lower dose of dichloromethane is needed to inhibit the proliferation of cancer cells by 50% compared to the methanol leaf extract.* In vitro* chemoprevention was also tested using the quinone reductase (QR) induction assay on the hepatoma (Hepa-1c1c7) cell line. The dichloromethane leaf extract induced significant QR activity, whereas the methanol leaf extract exhibited no significant inductive activity. These results indicate that the methanol extract of moringa leaves has better antioxidant activity, but the dichloromethane leaf extract has better anticancer activity on HepG2, Caco-2, and MCF-2 cells as well as better chemoprevention activity.

Khalafalla et al. (2010) [44] tested the anticancer effects of* M. oleifera* leaf extracts on primary leukemia cells harvested from 15 patients with acute myeloid leukemia (AML) and 10 patients with acute lymphoblastic leukemia (ALL). They also tested the effects of cold water, hot water, and 80% ethanol leaf extracts on the HepG2 cell line. The leaf extracts were first tested for their antioxidant activity using the DPPH assay. The hot water and 80% ethanol extracts had the highest antioxidant activity. They showed 63% and 77% inhibition of radical formation, respectively, compared to 49% inhibition by the cold water extract. The MTT assay was then performed to determine if the extracts could inhibit the proliferation of cancerous cells. The leaf extracts showed promising results, causing 72–82% of AML cells and 77–86% of ALL cells to die after 24 hours of incubation with 20 *µ*g/ml of the extract. After the same treatment, 69–81% of HepG2 cells died. The ethanol extract had the highest anticancer activity in both AML and ALL cells, followed by the hot water extract and then the cold water extract. For HepG2 cells, the hot water extract resulted in the strongest inhibition and the cold water extract had the weakest anticancer activity. Overall, the results showed that moringa leaf extracts had good anticancer activity* in vitro* against AML, ALL, and HepG2 cells.

### 3.3. In Vivo Anticancer Activity

Once* in vitro* studies show promising results, the next step is to determine whether the results can be replicated* in vivo*. Because humans are complex multicellular organism,* in vitro* results can only be used as indications of the anticancer activity of the moringa leaf extract.* In vivo* studies are required to move the research to the next level, as they can demonstrate the anticancer activity of the moringa leaf extract in a complex organism. Jung et al. (2015) [45] tested the effects of a water soluble moringa leaf extract* in vitro* and* in vivo* using a mouse model. The study started with tests of effects of moringa leaf extract anticancer activity on human hepatocellular carcinoma (HepG2) cells. The moringa leaf extract was extracted with cold water, and the HepG2 cells treated with leaf extract were tested by flow cytometry to determine the effects of the extract on DNA content and cell cycle stage of the cells. A dose-dependent increase of cells in the sub-G1 phase occurred as leaf extract concentration increased. The MTT assay also showed that cell proliferation was inhibited as leaf extract concentration increased. In the colony forming assay, cells treated with 50 *µ*g/ml of leaf extract (highest tested dose) showed a 70% reduction in colonies compared to the negative control. The Annexin V-fluorescein isothiocyanate (FITC)/PI flow cytometric assay showed that the apoptotic cell ratio was five times higher in the treatment group compared to the untreated control. Western blot analysis detected an increase in cleaved and deactivated PARP, which indicated that cells were dying due to accumulation of DNA damage. The levels of B-cell lymphoma-extra large (Bcl-xL) increased significantly. Because Bcl-xL is an antiapoptotic protein, its downregulation showed that more cells were undergoing apoptosis. The terminal deoxynucleotidyl transferase-mediated dUTP nick end labeling assay was performed to detect DNA strand breaks in apoptotic cells. The assay causes cells with DNA breaks and undergoing apoptosis to give off a bright green glow. As leaf extract concentration increased, the number of cells glowing and the intensity of the glow increased, indicating that more cells were undergoing apoptosis.

Based on these promising* in vitro *results, Jung et al. (2015) [45] conducted* in vivo* testing on immunodeficient nude mice using the hollow fiber assay. HepG2 and A549 cells were grown inside hollow fibers with 1 mm inner diameter. The fibers were then implanted into the subcutaneous cavity of the mice, and the mice were allowed to recover for three days before being fed leaf extract for four days. The positive control mice were given an intravenous injection of doxorubicin (an anticancer drug). After treatment, the fibers were recovered, and the cells were analyzed using the trypan blue exclusion assay, in which dead cells are stained blue. HepG2 cells were more susceptible to the moringa leaf extract than A549 cells. At the maximum tested dose of 200 mg/kg, the survival of HepG2 and A549 cells decreased by 60% and 50%, respectively. The moringa leaf extract decreased the survival of HepG2 cells to levels lower than those achieved by the doxorubicin control. These results illustrate the potential of using the moringa leaf extract as an anticancer drug.

Krishnamurthy et al. (2015) [46] conducted* in vitro* and* in vivo* studies to identify and characterize the potent anticancer fraction of the moringa leaf extract. The leaf extract was extracted with successive Soxhlet extractions using n-hexane, chloroform, ethyl acetate, and 50% methanol. The ethyl acetate leaf extract was further separated into 15 fractions with a silica gel column. The ethyl acetate and fraction 1 (F1) were subjected to standardization with HPTLC and qualitative phytochemical analysis. The HPTLC produced the distinct profile of the two extracts, and the phytochemical analysis identified steroid, flavonoid, and phenolic compounds in the ethyl acetate extract and steroids and phenolic compounds in F1. All of the different extracts and fractions were tested using the Hep2 human epidermoid cancer cell line to determine their anticancer activity. The cells were incubated with the extracts and fractions for 72 hours, and morphological changes were observed and noted every 24 hours. After 72 hours of incubation, the cells were fixed and stained with sulforhodamine B, which binds to cellular protein, to determine cell viability. Among all extracts and fractions tested, F1 had the best antiproliferative activity against Hep2 cells (IC_50_: 12.5 *µ*g/ml). The* in vivo* test was conducted in Swiss albino mice using F1. The acute oral toxicity of F1 on the mice was tested by giving them a 2000 mg/kg dose and observing them for 14 days before they were culled for gross necropsy analysis. Results indicated that F1 had very low toxicity, as no mice died and no abnormalities were observed in their internal organs. The IC_50_ of F1 was determined to be > 2000 mg/kg, which is category 5 in terms of acute toxicity which is relatively low in acute toxicity. The in vivo anticancer activity of F1 was tested using Dalton's lymphoma ascites (DLA) model. Mice were inoculated with the DLA via intraperitoneal injection and given treatment orally for 10 days. Mice given FI treatment had significantly longer survival compared to untreated mice and even slightly longer survival than mice treated with the anticancer drug 5-fluorouracil. This study showed that the F1 of the ethyl acetate moringa leaf extract had very high anticancer activity both* in vitro* and* in vivo*.

### 3.4. Mechanisms of Action

A number of researchers have also tried to identify the pathways and molecular changes involved in moringa leaf extract-induced cancer cell death. In a follow-up to Sreelatha and Padma's (2011) [40] study showing that the moringa leaf extract had cytotoxic effects on KB cells (HeLa contaminant cells), Sreelatha et al. (2011) [47] decided to do an in-depth study on the cytotoxic effects of moringa leaf extract on KB cells and tested the antiproliferative effects as well as induction of apoptosis by* M. oleifera* leaf extracts on cancerous KB cells. The MTT cell proliferation assay showed that 100 *µ*g/ml of the hot water leaf extract reduced cell viability by ~38% and increasing the concentration to 200 *µ*g/ml reduced cell viability by 60%. Thus, the leaf extract significantly inhibited KB cell proliferation in a dose-dependent manner. Some of the morphological changes observed in treated cells were cytoplasmic membrane shrinkage, loss of contact with neighboring cells, membrane blebbing, and apoptotic body formation, which are signs of cells undergoing apoptosis. Propidium iodide (PI) is a fluorescent intercalating agent that can bind to DNA and allows visualization of cell nuclei. PI can also be used as an index of the extent of apoptosis in cells because it is too big to pass through the intact cell membrane of living cells but is increasingly able to permeate cells undergoing apoptosis. When treated cells were stained with PI to detect any changes to the nuclei of live and dead cells, those treated with 200 *µ*g/ml of moringa leaf extract showed changes, including nuclear shrinkage, DNA condensation, and fragmentation. Thus, the extract appears to induce apoptosis in KB cells.

Sreelatha et al. (2011) [47] did further tests to quantify the apoptosis in the treated cells by determining the apoptotic index. Cells were treated with 4',6-diamidino-2-phenylindole (DAPI), which binds to the AT region of DNA to form a fluorescent complex and allows detection of abnormal nuclei, such as condensed or fragmented chromatin. Similar to PI, DAPI is more permeable to cells with compromised cell membranes, which is indicative of apoptosis. The cells treated with the leaf extract showed the presence of nuclear apoptotic bodies and chromatin condensation, which confirmed the PI results. In another test, treated cells were collected and their DNA isolated and analyzed by agarose gel electrophoresis to determine if their DNA was compromised. Cells treated with the leaf extract had fragmented DNA and produced a smear of DNA fragments on the gel. It is likely that apoptosis was caused by an increase in reactive oxygen species (ROS) in the mitochondria. The dichlorodihydrofluorescein diacetate (DCFH-DA) assay was performed to measure the levels of ROS in cells treated with the leaf extract. Cells treated with 200 *µ*g/ml of leaf extract showed a 350% increase in ROS compared to the negative control. This finding supports the hypothesis that an increase in ROS caused cell apoptosis in KB cells treated with moringa leaf extract. The study provides compelling evidence of the strong anticancer activity of moringa leaf extract against KB cells and indicates that KB cell death is achieved by increasing ROS levels in the cell and fragmenting cellular DNA.

In another study, Tiloke et al. (2013) [48] tested the effects of water soluble* M. oleifera* leaf extract on human alveolar epithelial cells derived from the lung cancer A549 cell line. The MTT assay showed that A549 cell viability decreased with increasing concentration of moringa leaf extract (IC_50_: 166.7 *µ*g/ml). This concentration of extract was used for subsequent tests. The thiobarbituric acid (TBARS) assay was used to gauge the ROS levels in the cells by measuring the levels of MDA produced. A549 cells treated with the IC_50_ concentration of leaf extract showed significantly higher levels of MDA compared to untreated controls (0.269 *µ*M versus 0.197 *µ*M). The antioxidant potential of the cells was also explored by measuring GSH (a strong antioxidant) level in the cells. Leaf extract-treated cells had lower levels of GSH (2.507 x 10^6^ RLU) compared to untreated cells (3.751 x 10^6^ RLU), which indicates that treated cells experienced higher oxidative stress, which depleted the GSH in the cells. DNA damage in the cells was assessed using single cell gel electrophoresis (i.e., the comet assay), which enables detection of DNA damage in individual eukaryotic cells. In this assay, the relative intensity of the comet tails to the comet head is an indication of the DNA damage in the cells: the longer the tail, the more the DNA breaks present, as DNA breakage allows the tails to become longer. In this assay, treated cells had significantly longer tails (18.52 *µ*m) compared to untreated cells (5.15 *µ*m). Next, Caspase-Glo® 3/7 and Caspase-Glo® 9 assays (Promega) were used to quantify the levels of caspase 3, 7, and 9 in the cells, which are responsible for apoptosis. Caspase 3/7 levels did not differ significantly between treated and untreated cells but treated cells had significantly higher levels of caspase 9 (~16.160 x 10^5^ RLU) compared to untreated cells (12.630 x 10^5^ RLU). The elevated caspase 9 indicates that treated cells have elevated apoptosis compared to untreated cells.

Tiloke et al. (2013) [48] next conducted Western blot analysis to evaluate how the moringa leaf extract affected proteins linked to cancer progression and apoptosis, including transcription factor nuclear factor-erythroid 2 p45-related factor 2 (Nrf2), which is responsible for activating expression of antioxidant proteins; tumor protein p53 (p53), which is a tumor suppressor that can cause apoptosis; second mitochondria-derived activator of caspases (SMAC/DIABLO), which binds inhibitor of apoptosis proteins (IAPs), thus freeing caspases to activate apoptosis; and poly [ADP-ribose] polymerase 1 (PARP-1), which is involved in DNA repair. Moringa leaf extract-treated cells showed reduced levels of Nrf2 and PARP-1, whereas p53 and SMAC/DIABLO protein levels increased. The decrease in Nrf2 corresponded with the lower levels of GSH in treated cells. The low levels of PARP-1 caused DNA damage to be insufficiently repaired, leading to increased DNA fragmentation which is consistent with the results of the comet assay showing increased DNA fragmentation. The increase in p53 and SMAC/DIABLO levels was consistent with what would be expected from cells undergoing apoptosis. Together these results indicate that treated cells experienced more apoptosis compared to untreated cells. When the mRNA expression levels of Nrf2 and p53 were further tested with quantitative polymerase chain reaction (qPCR), treated cells showed a 1.44-fold decrease in Nrf2 mRNA levels and a 1.59-fold increase in p53 mRNA levels when compared to untreated cells. The mRNA analysis confirmed that the leaf extract affected the expression of Nrf2 and p53 at the transcriptional level. Thus, moringa leaf extract induced apoptosis of A549 cells by disrupting the expression of proteins involved in antioxidant production, which increased the levels of ROS in the cells and led to increased DNA fragmentation and subsequent cell death.

Madi et al. (2016) [49] also investigated the mode of action of the anticancer activity of moringa leaf extract on the A549 lung cancer cell line. The leaf extract was prepared by soaking the dried leaf powder in hot water, and different concentrations of leaf extract based on the ratio of leaf powder to water were prepared (0.1% to 2.5%). Cell viability of extract-treated A549, HepG2, CaCo2, Hek293, and Jurkat cells was measured and compared. Although the leaf extract caused dose-dependent reduction in viability of all tested cell types, some were more sensitive than others. A549 cells were the most susceptible to the leaf extract (IC_50_: 0.05%) compared to the other cell lines (IC_50_: 0.1–0.4%). The ATP bioluminescence assay revealed a significant decrease in ATP levels with increasing moringa leaf extract concentration, which indicates that treatment resulted in fewer live cells, as ATP is required for live and active cells. The p-nitro-blue-tetrazolium salt assay showed a significant increase in ROS levels with increased concentration of the leaf extract. Elevated levels of ROS cause DNA damage, which leads to cell death. The ApoGSH colorimetric test showed a significant decrease in GSH level with increasing extract concentration. The decrease of GSH together with the increase in ROS and decrease in ATP with increasing extract concentration suggests that the leaf extract compromises the mitochondrial pathway of the cell to induce cell death. Measurement of the mitochondrial membrane potential using cell-permeable lipophilic JC-1 staining showed that the leaf extract induced mitochondrial membrane potential depolarization. In just one hour of 0.05% moringa treatment, a noticeable induction in mitochondrial membrane depolarization was observed.

Madi et al. (2016) [49] next used Western blot analysis to determine if there were any changes in protein expression of the apoptotic markers p53, apoptotic inducible factor (AIF), cytochrome c, and SMAC/DIABLO. All of these apoptotic markers showed increased expression in treated cells, which indicates higher levels of apoptosis in the cells. To verify the increase in cell death, lactate dehydrogenase (LDH) levels were measured. LDH is released when the cell membranes are compromised, for example, during cell death or cell membrane damage. A549 cells treated with leaf extract had elevated levels of LDH, which indicates an increase in cell death. Finally, apoptosis in cells was measured using the FLICA assay, which is an immunofluorescent detection method that stains caspase proteins that are produced to activate apoptosis in cells. After 24 hours of treatment with 0.05% moringa leaf extract, many cells fluoresced, indicating the presence of activated caspase and the activation of apoptosis in the cells. Based on the results of these experiments, Madi et al. (2016) postulated that the moringa leaf extract induces apoptosis in A549 cells via depolarization of the mitochondrial membrane, leading to a decrease in ATP, which in turn causes an increase in ROS levels, which causes more depolarization of the membrane. The positive feedback loop causes ROS levels to further increase and decreases levels of the antioxidant GSH and eventually causes the cells to undergo apoptosis.

Berkovich et al. (2013) [50] tested the effects of the water soluble leaf extract of* M. oleifera* on the pancreatic cancer cells lines Panc-1, COLO-357, and p34. Moringa leaf powder was added to boiling water and soaked for 5 minutes before being filtered and stored at 4°C before use. The leaf extract was tested for its effects on cell viability using the 2,3-Bis-(2-Methoxy-4-Nitro-5-Sulfophenyl)-2H-Tetrazolium-5-Carboxanilide (XTT) assay, which is like the more commonly used MTT assay. The extract had an inhibitory effect on cell proliferation of all cell lines, with Panc-1 cells being the most susceptible to treatment (IC_50_: 1.1 mg/ml). p34 was the second most susceptible (IC_50_: 1.5 mg/ml), followed by COLO-357 (IC_50_: 1.8 mg/ml). The Panc-1 cell line was further tested because it was most sensitive to the moringa leaf extract. First the cells were analyzed with flow cytometry (FACS) to evaluate the effects of the leaf extract on the cell cycle of treated cells. Treated and control cells were stained with PI to detect changes in the cell nucleus. Treated cells showed high proportions of cells in the sub-G1 phase, which increased with increasing concentration of leaf extract. Trypan blue staining, which stains dead cells, showed that moringa leaf extract induced significant cytotoxicity in cells treated with 0.75 mg/ml of extract, with 30% of cells dead compared to the untreated control.

Berkovich et al. (2013) [50] continued the study with Western blot analysis of Panc-1 cells to detect levels of proteins of the nuclear factor kappa B (NF-kB) signalling pathway. NF-kB is a proinflammatory transcription factor, and its signalling pathway reportedly plays a significant role in the resistance of pancreatic cancer cells to apoptosis-based chemotherapy. Western blot analysis was used to measure the levels of proteins p65, p-IkB*α*, and IkB*α* which are involved in the pathway. Cells treated with 0.25 mg/ml of leaf extract showed a significant reduction of these proteins compared to untreated cells, which indicates that the moringa leaf extract can downregulate the NF-kB pathway in Panc-1 cells. The final test was to treat Panc-1 cells with moringa leaf extract in conjunction with a conventional anticancer drug, cisplatin, to determine if their interactions were additive, antagonistic, or synergistic. The Western blot results suggested that the leaf extract could sensitise the cancer cells to chemotherapeutic drugs, so the researchers expected a synergistic effect and therefore utilized a low inhibitory dose of cisplatin and leaf extract. The viability of treated cells was determined with the XTT assay. The results supported the Western blot results, as the treatment showed synergistic effects with the calculated combination index (CI) ranging from 0.156 to 0.652. A CI of < 1 indicates synergistic effects, with values closer to zero indicating higher synergistic effects. Based on these results, Berkovich et al. (2013) concluded that moringa leaf extract had cytotoxic effects against the Panc-1, COLO-357, and p34 cell lines. This study shows that not only is the moringa leaf extract cytotoxic to Panc-1 cells, but there is a possibility of using it in conjunction with cisplatin to vastly improve the effectiveness of the drug.

Jung (2014) [51] tested the effects of moringa leaf extract on COS-7 cells (African green monkey kidney cells, which are normal, noncancerous cells) and A549 lung cancer cells. The moringa leaf extract was extracted with cold water and lyophilized for dissolution into working concentrations before use. The MTT assay showed a reduction of 75% of A549 cells when treated with 200 *µ*g/ml of leaf extract. However, COS-7 cells showed only mild cytotoxicity when treated with 200 *µ*g/ml of leaf extract. The COS-7 cells treated with 600 *µ*g/ml of leaf extract still have cell viability of more than 50%. Thus, the leaf extract was highly cytotoxic to cancerous A549 cells but not to normal COS-7 cells. Jung (2014) [51] also tested the cytotoxic effects of moringa leaf extract on lung cancer cells (H23 and H358), breast cancer cells (MCF-7), epidermoid carcinoma cells (A431), and fibro-sarcoma (HT1080) cells and found dose-dependent cytotoxic effects on all of the tested cells. Flow cytometry analysis of extract-treated A549 cells revealed a dose-dependent increase of cells in the sub-G1 phase of the cell cycle. At 200 *µ*g/ml, 21% of cells were in the sub-G1, whereas the value was 93% at 400 *µ*g/ml. The colony formation assay, used to test the effects of the leaf extract on clonogenicity of A549 cells, showed that colony formation was reduced by 50% when A549 cells were treated with 50 *µ*g/ml of leaf extract, and the value dropped to 0% when treated with 100 *µ*g/ml of leaf extract. This result shows that the moringa leaf extract has very high anticlonogenic effects on A549 cells. DCFH-DA analysis showed a significant decrease in ROS concentrations in treated A549 cells, indicating that the moringa leaf extract also has free radical scavenging abilities.

Next the effects of the leaf extract on the expression of proteins and messenger RNA (mRNA) of A549 cells were investigated with Western blot analysis and RT-PCR. Most proteins and mRNA tested showed a dose-dependent decrease. The levels of proteins tested showed a significant decrease with increasing leaf extract, although some proteins exhibited increased expression (e.g., heat shock proteins). Jung then conducted a gene microarray of 23,753 genes. Base on the results only 0.9% of genes were upregulated by 2-fold, whereas 90.9% were downregulated. Overall, the mRNA results were similar to the protein results, which suggests that protein expression decreased through mRNA downregulation. Further analysis of ribosomal RNA (rRNA) showed significant degradation of rRNA in the 200 *µ*g/ml and 300 *µ*g/ml moringa leaf extract treatment groups. Together, these results indicate that A549 cells treated with moringa leaf extract experienced translational problems; as rRNA was degraded, heat shock proteins were upregulated to help deformed proteins acquire their correct configuration. These results suggest that the moringa leaf extract causes cell death in A549 cells by compromising the gene translation process in the cells.

### 3.5. Radioprotective Effects

Rao et al. (2001) [52] described the radioprotective effects of the* M. oleifera* leaf extract* in vivo*. They used 50% methanol and a Soxhlet setup to obtain the moringa leaf extract. The acute toxicity of the extract was determined in Swiss albino mice. A high dose of the extract was administered to the mice intraperitoneally. The LD_50_ (dose to kill 50% of animals within 72 hours) was 7.42 g/kg, which is very high and indicates that the leaf extract has very low toxicity. With the upper limit of the testing dose determined, a dose response study of the radioprotective effects of the moringa leaf extract was performed. Mice were given various amounts of leaf extract ranging from 10 to 200 mg/kg intraperitoneally before being exposed to a lethal dose of 11 Gy gamma radiation. The survival of mice over 30 days then was observed. Mice given 150 mg/kg of leaf extract had maximum survival, and 200 mg/kg of leaf extract did not increase the survival rate. Therefore, the 150 mg/kg dose was selected to study protection of bone marrow chromosome against radiation.

The mice were given intraperitoneal injections of the leaf extract one hour before being exposed to 4 Gy of whole body gamma irradiation. The mice were given either a single dose of 150 mg/kg or a fractionized dose of 30 mg/kg over 5 days, with the last 30 mg/kg dose administered one hour before the radiation treatment. The mice were analyzed on days 1, 2, and 7; one femur was used for chromosome analysis and the other was used for micronucleus count. The chromosome analysis was performed to observe aberrations in the bone marrow cells such as chromosome breaks, fragments, rings, and dicentrics. The micronucleus count was conducted to determine the amount of micronucleated polychromatic erythrocytes (MPEs) and micronucleated monochromatic erythrocytes (MMEs) present. The micronuclei in both MPEs and MMEs are whole chromosomes or chromosome fragments that do not fully separate during mitosis and thus are a good measure of genotoxic effects of a treatment. The single 150 mg/kg leaf extract dose caused a significant reduction in both chromosome aberrations and MPEs and MMEs over the test period compared to irradiated mice without any treatment. The fractionized delivery of the leaf extract resulted in even better radioprotective effects on the mice, as the chromosome aberrations and levels of MPEs and MMEs were even lower than those in mice given the single 150 mg/kg dose. The antioxidant properties of the moringa leaf extract were also studied using the Fenton reaction and measurement of the levels of TBARS. Dose-dependent inhibition of TBARS formation was detected for the moringa leaf extract, indicating that it has good antioxidant activity. This study illustrates the possibility of utilizing moringa leaf extract as a radioprotective agent to prevent cancer formation.

## 4. Anticancer Activity of Extracts from Other Parts of* M. oleifera*: Bark, Seeds, and Roots

Researchers have also assessed the anticancer activities of other parts of the moringa tree, including the bark, seeds, and roots. Al-Asmari et al. (2015) [53] tested the effects of leaf, seed, and bark ethanol extracts of* M. oleifera *on ileocecal adenocarcinoma (HCT-8) and human breast adenocarcinoma from breast mammary glands (MDA-MB-231) cell lines. Results of the motility test to look for phenotypic changes caused by a 500 *µ*g/ml dose of the extracts revealed reduction of motility by 90% for the leaf extract, 50% for the bark extract, and no detectable decrease for the seed extract. The* in vitro* clonogenic survival assay showed an 80–90% reduction in colony formation for the leaf and bark extracts but no significant reduction for the seed extract. Results of the cell viability assay also showed significant cell death caused by the leaf and bark extracts at 250 *µ*g/ml and toxic effects on the cells at 500 *µ*g/ml but no significant effects for the seed extract. Al-Asmari et al. (2015) then tested the cell lines for apoptosis using the Annexin V and dead cell kit. Leaf and bark extracts induced late apoptosis in a dose dependant manner, whereas the seed extract had no noticeable effects on the cells. Finally, the cell cycle assay showed G2/M enrichment in MDA-MB-231 cells, at 29% in the control, 53% in the leaf extract treatment, and 61% in the bark extract treatment, compared to HCT-8 cells (13%, 38%, and 33%, respectively). The seed extract did not have significant effects on the cell cycle in either cell type. G2/M enrichment is an indication that cell cycle arrest is occurring in the cell, thereby inhibiting cell division.

Guevara* et al.* (1996) [54] studied the anti-inflammatory and antitumor properties of the seed extract of* M. oleifera*. The crude seed extract of dried and green seeds was extracted with ethanol, concentrated, and then partitioned into hexane, ethyl acetate, butanol, and water extracts. The different fractions were tested for their anti-inflammatory activity* in vivo* on mice, which had carrageenan-induced inflammation in the hind paw. The crude ethanol extract of the dried seeds reduced the inflammation by 85% at the dosage of 3 mg/g body weight, whereas that of mature green seeds reduced inflammation by 77%. The hexane fraction of dried seeds yielded the same result, whereas the butanol and water fractions of dried seeds reduced inflammation by only 34%. In contrast, the ethyl acetate fraction of dried seeds caused a 267% increase in inflammation, and the mice died after oral administration. To test for antitumor activity, the extracts were tested for their ability to inhibit the formation of Epstein-Barr virus-early antigen (EBV-EA) induced by 12-0 tetradecanoylphorbol-13-acetate (TPA). The crude ethanol extract inhibited EBV-EA formation by 100% at the dose of 100 *µ*g/ml. Overall, the* M. oleifera* seed ethanol extract showed good anti-inflammatory activity and may be a potential antitumor agent.

Rajesh et al. (2012) [55] tested the anticancer activity of the moringa seed methanol extract on A549, Hep-2 (HeLa contaminant carcinoma), 502713 HT-29 (colon cancer), and IMR-32 (neuroblastoma) cell lines. They used the sulforhodamine B (SRB) assay to determine the antiproliferative effects of the moringa seed extract. SRB staining is an indirect measure of cells because it stains cellular proteins, and the absorbance of the SRB-stained cell suspension is used to determine the number of cells in solution. A dose of 100 *µ*g/ml of seed extract was applied to each of the cell lines, and cells were incubated for 48 hours before SRB staining was performed. The results showed high antiproliferative activity of the extract for A549 cells (80% inhibition), 502713 HT-19 cells (95% inhibition), and IMR-32 cells (93% inhibition). However, the seed extract did not inhibit the growth of Hep-2 cells. Thus, the seed extract exhibited anticancer activity against certain cell lines but not all of them.

Guevara et al. (1996) [56] isolated the active compounds from the* M. oleifera* seed extract to test its anticancer properties* in vitro* and* in vivo*. The ethanol extract was partitioned with CCl_4_ and H_2_O, and the CCl_4_ fraction was subjected to flash column chromatography to isolate the different active compounds in the fraction. The active compounds isolated were *β*-sitosterol, glycerol-1-(9-octadecanoate), 3-O-(6-O-oleoyl-*β*-D-glucopyranosyl)-*β*-sitosterol, and *β*-sitosterol-3-O-glucopyranoside. A few fractions of isolates were then further separated with HPLC to obtain O-ethyl-4-a-L-rhamnosyloxy benzyl carbamate, 4-(*α*-L-rhamnosyloxy) benzyl isothiocyanate, niazimicin, and niazirin. All of the isolates were verified with IR, NMR, and MS data. The compounds were first screened for anticancer activity* in vitro* with EBV genome-carrying lymphoblastoid cells (i.e., Raji cells). Treatment of the Raji cells with n-butyric acid and tetradecanoylphorbol acetate activated the EBV genome so that the cells produced EBV-EA. The induced Raji cells were treated with the different isolated compounds to determine the effectiveness of the compounds in preventing induction of the EBV genome. The most effective compounds were *β*-sitosterol-3-O-glucopyranoside (IC_50_: 27.9 *µ*g/ml), 4-(*α*-L-rhamnosyloxy) benzyl isothiocyanate (IC_50_: 32.7 *µ*g/ml), and niazimicin (IC_50_: 35.3 *µ*g/ml). Due to limited availability, only niazimicin was tested* in vivo* for its inhibition of tumor promotion. The test was conducted on pathogen-free IRC mice treated with 7,12-Dimethylbenz(a)anthracene (DMBA) to induce tumor growth, and TPA was applied to the skin to promote tumor growth twice a week. For the treated mice, 85 nmol of niazimicin was applied to the skin of the mice one hour before application of TPA. The incidence of papillomas was observed weekly for 20 weeks. Compared to untreated mice, niazimicin treatment delayed the promotion of tumors by 50%. Niazimicin also reduced the number of papillomas on the mice that did get papillomas compared to untreated mice. These results indicate that the moringa seed extract contains the active compound niazimicin that has very high antitumor promoter activities and can delay the activation of tumor formation.

Costa-Lotufo et al. (2005) [57] tested 11 plants that are extensively used in Bangladeshi folk medicine to determine their anticancer potential. The plants were tested using the brine shrimp, sea urchin embryo, MTT, and hemolytic assays. The MTT assay was conducted on murine melanoma (B-16), human colon carcinoma (HCT-8), and leukemia (CEM and HL-60) cell lines. Moringa roots were extracted with absolute ethanol using a cold extraction process. The crude extract was further partitioned into n-hexane, chloroform, methanol, and water fractions. The extract did not show any cytotoxicity in the brine shrimp assay, did not cause bursting of red blood cells in the hemolytic assay, and had only moderate efficacy in inhibiting sea urchin embryo formation. It did show good anticancer activity on multiple cell lines when tested with the MTT assay. The extract inhibited the growth of CEM (IC_50_: 12.7 *µ*g/ml) and B-16 (IC_50_: 28.8 *µ*g/ml) cells. Out of the 11 plants tested, only 3 had anticancer potential:* Oroxylum indicum, M. oleifera*, and* Aegle marmelos.* The results of the study showed that the moringa root extract has great anticancer potential, and further tests should be conducted to verify the result.

## 5. Conclusion

The studies reviewed herein show that different parts of the* M. oleifera* plant have antioxidant, anti-inflammatory, anticancer, chemopreventive, and even radioprotective activities. The active compounds identified to contribute to these activities are listed in Table 1. Most of the research has focused on moringa leaf extracts, and the published results suggest that the extract is cytotoxic to a wide variety of different cancerous cells. The leaf extract appears to have relatively low toxicity against normal cells, and very low toxicity was detected when the extract was administered orally during* in vivo* testing on mice and rats. There are limitations to the studies, however. For example, different studies used a variety of different extraction techniques and solvents, which might account for some of the discrepancies in the results due to different amounts of active compounds in the final extract. More studies are needed to identify any differences in anticancer activity caused by the different extraction techniques and solvents. Although differences in results were found, numerous studies also reported similar findings, especially studies regarding the pathways by which the moringa leaf extract leads to cancer cell apoptosis. Several studies reported that the leaf extract causes elevated levels of ROS in the cells, which leads to DNA damage and eventually apoptosis. Overall, the studies described herein illustrate that* M. oleifera* extracts have huge potential to be developed into an anticancer drug. However, more research and development are required to move forward.

## Figures and Tables

**Table 1:** Active compounds of extracts from different parts of the moringa plant that contribute to the anticancer activities base on in vitro and in vivo studies.

**Parts of Moringa **	**Extraction method**	**Experiments**	**Active compounds**	**Citation**
Leaf	Methanol, ethanol, ethyl acetate and chloroform extracts	Cytotoxicity test on U266B1 cells.	Flavonoid and alkaloid group similar to vincristine and vinblastine.	Parvathy & Umamaheshwari (2007) [41]
	Hot water, methanol and hexane extracts	Cytotoxicity test on HeLa cells	Aqueous extract has best anticancer activity.	Nair & Varalakshmi (2011) [42]
	Methanol and dichloromethane extracts	Cytotoxicity test on HepG2, Caco-2 and MCF-7 cells. Quinone reductase induction assay on Hepa-1c1c7	Dichloromethane extract has best cytotoxic and chemopreventive activity. Active compounds are quercetin, kaempferol, glucosinolate and sulforaphane.	Charoensin (2014) [43]
	Cold water, hot water and 80% ethanol extracts	Cytotoxicity test on AML, ALL and HepG2 cells	Ethanol extract has the best cytotoxicity against AML and ALL. Hot water extract is most cytotoxic towards HepG2 cells. Active compounds are phenolic compounds, especially glycosides.	Khalafalla et al. (2010) [44]
	Cold water extract	Cytotoxicity test on HepG2 cells. In vivo study using hollow fibre assay on HepG2 and A549 cells	Active compounds are water soluble bioactive compounds.	Jung et al. (2015) [45]
	Successive extraction with n-hexane, chloroform, ethyl acetate and 50% methanol. Ethyl acetate extract was further separated into 15 fractions	Cytotoxicity test on HepG2 cells. In vivo studies to determine toxicity. In vivo study with Dalton's lymphoma ascites (DLA) model	Fraction 1 (F1) from ethyl acetate was the most cytotoxic against HepG2. Active compounds are steroids and phenolic compounds.	Krishnamurthy et al. (2015) [46]
	Hot water extract	Cytotoxic test on KB cells.	Active compounds are polyphenols primarily quercetin and kaempferol.	Sreelatha et al (2011) [47]
	Hot water extract	Cytotoxic test on A549 cells.	Active compounds are glucosinolates, isothiocyanates, niazimicin, niaziminin quercetin, thiocarbamate, carbamates and nitrile glycosides.	Tiloke et al. (2013) [48]
	50% ethanol extract	In vivo study on Swiss albino mice to test radioprotective effects	Active compound is Vitamin C.	Rao et al. (2001) [52]
	Ethanol extract	Cytotoxic test on HCT-8, MDA-MB-231	Active compounds are D-allose and hexadecanoic acid.	Al-Asmari et al. (2015) [53]

Seed	Dried and green seeds ethanol extract partitioned into hexane, ethyl acetate, butanol, and water.	In vivo study of anti-inflammation activity. Antitumor activity tested on ability to inhibit the formation of EBV-EA induced by TPA.	Ethanol extract has best anti-inflammation and antitumor activity.	Guevara *et al.* (1996) [54]
	Methanol extract	Cytotoxic test on A549, Hep-2,HT-29, and IMR-32.	Active compounds are alkaloids.	Rajesh et al. (2012) [55]
	Ethanol extract whereby the active compounds were isolated with flash column chromatography and further isolated with HPLC.	Tested anticancer activity in vitro with EBV genome-carrying lymphoblastoid cells, Raji cells. Niazimicin tested on mice induced to form tumours.	The active compounds which prevent induction of EBV genome are: *β*-sitosterol-3-O-glucopyranoside, 4- (*α*-L-rhamnosyloxy) benzyl isothiocyanate and niazimicin. Niazimicin was able to delay the formation of tumours and reduce the number of tumours in the *in vivo *study.	Guevara et al. (1999) [56]

Bark	Ethanol extract	Cytotoxic test on HCT-8, MDA-MB-231	Active compounds are Isothiocyanate, hexadecanoic acid and eugenol	Al-Asmari et al. (2015) [53]

## References

[1] Paliwal R., Sharma V. (2011). A Review on Horse Radish Tree (Moringa oleifera): A Multipurpose Tree with High Economic and Commercial Importance. *Asian Journal of Biotechnology*.

[2] Radovich T. Farm and Forestry Production and Marketing Profile for Moringa (Moringa oleifera) groforestry.net/images/pdfs/Moringa_specialty_crop.pdf Ted Radovich.

[3] Nayar P. K. *Retiring? Try the Fidel Stick-Indian drumstick revolution in Castro’s residence*.

[4] Beaulah A., Vadivel E., Rajadurai K. R. Effect of organic and inorganic fertilizers on growth characters of Moringa (Moringa oleifera Lam.) cv. PKM 1. SOUTH INDIAN HORTICULTURE, 52(1/6), 183. Effect of organic and inorganic fertilizers on growth characters of moringa (Moringa oleifera Lam.) cv. PKM 1.

[5] Thurber M. D., Fahey J. W. (2009). Adoption of Moringa oleifera to combat under-nutrition viewed through the lens of the "Diffusion of innovations" theory. *Ecology of Food and Nutrition*.

[6] Banerji R., Verma S. C., Pushpangadan P. (2003). Oil potential of moringa. *Natural Product Radiance*.

[7] Ojiako E. N., Okeke C. C. (2013). Determination of antioxidant of Moringa oleifera seed oil and its use in the production of a body cream. *Asian Journal of Plant Science and Research*.

[8] Amagloh F. K., Benang A. (2009). Effectiveness of *Moringa oleifera* seed as coagulant for water purification. *African Journal of Agricultural Research*.

[9] Ghebremichael K. A. (2004). *Moringa seed and pumice as alternative natural materials for drinking water treatment [Ph.D. thesis]*.

[10] Mridha M., Al-Barakah F. (2017). Green cultivation of moringa on arid agricultural land in Saudi Arabia. *Acta Horticulturae*.

[11] Ashfaq M., Basra S. M. A., Ashfaq U. (2012). Moringa, a miracle plant for agro-forestry. *Journal of Agriculture and Social Sciences*.

[12] Ganguly S. (2013). Indian ayurvedic and traditional medicinal implications of indigenously available plants, herbs and fruits: A review. *International Journal of Research in Ayurveda and Pharmacy*.

[13] Agarwal V. (2017). *The Magical Moringa*.

[14] Anwar F., Latif S., Ashraf M., Gilani A. H. (2007). *Moringa oleifera*: a food plant with multiple medicinal uses. *Phytotherapy Research*.

[15] Nandave M., Ojha S. K., Joshi S., Kumari S., Arya D. S. (2009). *Moringa oleifera* leaf extract prevents isoproterenol-induced myocardial damage in rats: Evidence for an antioxidant, antiperoxidative, and cardioprotective intervention. *Journal of Medicinal Food*.

[16] Pari L., Kumar N. A. (2002). Hepatoprotective activity of *Moringa oleifera* on antitubercular drug-induced liver damage in rats. *Journal of Medicinal Food*.

[17] Gilani A. H., Aftab K., Suria A. (1994). Pharmacological studies on hypotensive and spasmolytic activities of pure compounds from *Moringa oleifera*. *Phytotherapy Research*.

[18] Morton J. F. (1991). The horseradish tree, *Moringa pterygosperma* (Moringaceae)—a boon to arid lands?. *Economic Botany*.

[19] Ghasi S., Nwobodo E., Ofili J. O. (2000). Hypocholesterolemic effects of crude extract of leaf of Moringa oleifera Lam in high-fat diet fed wistar rats. *Journal of Ethnopharmacology*.

[20] Pal S. K., Mukherjee P. K., Saha B. P. (1995). Studies on the antiulcer activity of Moringa oleifera leaf extract on gastric ulcer models in rats. *Phytotherapy Research*.

[21] Faizi S., Siddiqui B. S., Saleem R., Aftab K., Shaheen F., Gilani A.-U. (1998). Hypotensive constituents from the pods of Moringa oleifera. *Planta Medica*.

[22] Gupta R., Mathur M., Bajaj V. K. (2012). Evaluation of antidiabetic and antioxidant activity of *Moringa oleifera* in experimental diabetes. *Journal of Diabetes*.

[23] Zaffer M., Ahmad S., Sharma R., Mahajan S., Gupta A., Agnihotri R. K. (2014). Antibacterial activity of bark extracts of Moringa oleifera Lam. against some selected bacteria. *Pakistan Journal of Pharmaceutical Sciences*.

[24] Caceres A., Saravia A., Rizzo S., Zabala L., De Leon E., Nave F. (1992). Pharmacologic properties of Moringa oleifera. 2: screening for antispasmodic, antiinflammatory and diuretic activity. *Journal of Ethnopharmacology*.

[25] Ruckmani K., Kavimani S., Anandan R., Jaykar B. (1998). Effect of *Moringa oleifera* lam on paracetamol-induced hepatotoxicity. *Indian Journal of Pharmaceutical Sciences*.

[26] Randriamboavonjy J. I., Loirand G., Vaillant N. (2016). Cardiac protective effects of moringa oleifera seeds in spontaneous hypertensive rats. *American Journal of Hypertension*.

[27] Shukla S., Mathur R., Prakash A. O. (1988). Antifertility profile of the aqueous extract of Moringa oleifera roots. *Journal of Ethnopharmacology*.

[28] WHO (2017). *Cancer Fact Sheet*.

[29] Newman D. J., Cragg G. M., Snader K. M. (2003). Natural products as sources of new drugs over the period 1981–2002. *Journal of Natural Products*.

[30] Wall M. E., Wani M. C., Cook C. E., Palmer K. H., McPhail A. T., Sim G. A. (1966). Plant antitumor agents. I. The isolation and structure of camptothecin, a novel alkaloidal leukemia and tumor inhibitor from Camptotheca acuminata. *Journal of the American Chemical Society*.

[31] Johnson I. T. (2007). Phytochemicals and cancer. *Proceedings of the Nutrition Society*.

[32] Awodele O., Oreagba I. A., Odoma S., Teixeira Da Silva J. A., Osunkalu V. O. (2012). Toxicological evaluation of the aqueous leaf extract of Moringa oleifera Lam. (Moringaceae). *Journal of Ethnopharmacology*.

[33] Siddhuraju P., Becker K. (2003). Antioxidant properties of various solvent extracts of total phenolic constituents from three different agroclimatic origins of drumstick tree (*Moringa oleifera* Lam.) leaves. *Journal of Agricultural and Food Chemistry*.

[34] Amaglo N. (2006). *How to Produce Moringa Leaves Efficiently?*.

[35] Sankhalkar S., Vernekar V. (2016). Quantitative and Qualitative analysis of Phenolic and Flavonoid content in Moringa oleifera Lam and Ocimum tenuiflorum L.. *Pharmacognosy Research*.

[36] Reuter S., Gupta S. C., Chaturvedi M. M., Aggarwal B. B. (2010). Oxidative stress, inflammation, and cancer: How are they linked?. *Free Radical Biology & Medicine*.

[37] Verma A. R., Vijayakumar M., Mathela C. S., Rao C. V. (2009). In vitro and in vivo antioxidant properties of different fractions of Moringa oleifera leaves. *Food and Chemical Toxicology*.

[38] Alam M. N., Bristi N. J., Rafiquzzaman M. (2013). Review on *in vivo* and *in vitro* methods evaluation of antioxidant activity. *Saudi Pharmaceutical Journal*.

[39] Al-Yahya M., Mothana R., Al-Said M. (2012). Attenuation of CCl4-induced oxidative stress and hepatonephrotoxicity by Saudi Sidr honey in rats. *Evidence-Based Complementary And Alternative Medicine*.

[40] Sreelatha S., Padma P. R. (2011). Modulatory effects of *Moringa oleifera* extracts against hydrogen peroxide-induced cytotoxicity and oxidative damage. *Human & Experimental Toxicology*.

[41] Parvathy M. V. S., Umamaheshwari A. (2007). Cytotoxic effect of *Moringa oleifera* leaf extracts on human multiple myeloma cell lines. *Trends in Medical Research*.

[42] Nair S., Varalakshmi K. (2011). Anticancer, cytotoxic potential of Moringa oleifera extracts on HeLa cell line. *Journal of Natural Pharmaceuticals*.

[43] Suphachai C. (2014). Antioxidant and anticancer activities of Moringa oleifera leaves. *Journal of Medicinal Plants Research*.

[44] Khalafalla M. M., Abdellatef E., Dafalla H. M. (2010). Active principle from *Moringa oleifera* Lam leaves effective against two leukemias and a hepatocarcinoma. *African Journal of Biotechnology*.

[45] Jung I. L., Lee J. H., Kang S. C. (2015). A potential oral anticancer drug candidate, Moringa oleifera leaf extract, induces the apoptosis of human hepatocellular carcinoma cells. *Oncology Letters*.

[46] Krishnamurthy P. T., Vardarajalu A., Wadhwani A., Patel V. (2015). Identification and characterization of a potent anticancer fraction from the leaf extracts of moringa oleifera L. *Indian Journal of Experimental Biology (IJEB)*.

[47] Sreelatha S., Jeyachitra A., Padma P. R. (2011). Antiproliferation and induction of apoptosis by *Moringa oleifera* leaf extract on human cancer cells. *Food and Chemical Toxicology*.

[48] Tiloke C., Phulukdaree A., Chuturgoon A. A. (2013). The antiproliferative effect of Moringa oleifera crude aqueous leaf extract on cancerous human alveolar epithelial cells. *BMC Complementary and Alternative Medicine*.

[49] Madi N., Dany M., Abdoun S., Usta J. (2016). Moringa oleifera's Nutritious Aqueous Leaf Extract Has Anticancerous Effects by Compromising Mitochondrial Viability in an ROS-Dependent Manner. *Journal of the American College of Nutrition*.

[50] Berkovich L., Earon G., Ron I., Rimmon A., Vexler A., Lev-Ari S. (2013). Moringa Oleifera aqueous leaf extract down-regulates nuclear factor-*κ*B and increases cytotoxic effect of chemotherapy in pancreatic cancer cells. *BMC Complementary and Alternative Medicine*.

[51] Jung I. L. (2014). Soluble extract from *Moringa oleifera* leaves with a new anticancer activity. *PLoS ONE*.

[52] Rao A. V., Uma Devi P., Kamath R. (2001). In vivo radioprotective effect of Moringa oleifera leaves. *Indian Journal of Experimental Biology (IJEB)*.

[53] Al-Asmari A. K., Albalawi S. M., Athar M. T., Khan A. Q., Al-Shahrani H., Islam M. (2015). Moringa oleifera as an anti-cancer agent against breast and colorectal cancer cell lines. *PLoS ONE*.

[54] Guevara A. P., Vargas C., Milagros U. Y. (1996). Anti-inflammatory and antitumor activities of seed extracts of malunggay, Moringa oleifera L. Moringaceae. *Philippine Journal of Science*.

[55] Rajesh A. S., Kiran N. S. S., Tripathi P. C., Verma K. (2012). In vitro cytotoxicity of Moringa oleifera against different human cancer cell lines. *Asian Journal of Pharmaceutical and Clinical Research*.

[56] Guevara A. P., Vargas C., Sakurai H. (1999). An antitumor promoter from *Moringa oleifera* Lam.. *Mutation Research - Genetic Toxicology and Environmental Mutagenesis*.

[57] Costa-Lotufo L. V., Khan M. T. H., Ather A. (2005). Studies of the anticancer potential of plants used in Bangladeshi folk medicine. *Journal of Ethnopharmacology*.

